# Correction to: Triple Loss of Function of Protein Phosphatases Type 2C Leads to Partial Constitutive Response to Endogenous Abscisic Acid

**DOI:** 10.1093/plphys/kiad681

**Published:** 2024-01-03

This is a correction to: Silvia Rubio, Americo Rodrigues, Angela Saez, Marie B. Dizon, Alexander Galle, Tae-Houn Kim, Julia Santiago, Jaume Flexas, Julian I. Schroeder, Pedro L. Rodriguez, Triple Loss of Function of Protein Phosphatases Type 2C Leads to Partial Constitutive Response to Endogenous Abscisic Acid, Plant Physiology, Volume 150, Issue 3, July 2009, Pages 1345–1355, https://doi.org/10.1104/pp.109.137174

In the originally published version of this manuscript, duplication was present in Figure 4C.

The image in the plate of figure 4C labelled as pp2ca-1 contains seedlings duplicated from the plate that contains hab1-1abi1-2 seedlings. Red boxes shown below in Figure 4C indicate duplication from the hab1-1abi1-2 seedlings. Figure 4C was generated in the last author's laboratory. Authors apologize that this problem was not detected during the preparation of the manuscript.

The pp2ca-1 image should represent a known ABA hypersensitive control with the single *pp2ca* mutant. The new mutants described in this article in 2009, particularly the triple pp2c mutants, have been widely used by the ABA research community and their phenotype has been reproduced in different laboratories.

Additionally, new repeat experiments described below indicate that the main findings of the work (physiological characterization of double and triple pp2c mutants) are reliable.

Given that the pp2ca-1 image affects germination data of figure 4B for the pp2ca mutant, the authors repeated this set of experiments. The authors here provide data and source files for the results of these experiments, which reproduced the results for the pp2c double and triple mutants, as reported previously in Rubio et al., 2009. This includes the ABA hypersensitivity of the pp2ca mutant. A corrected figure 4B, given below, provides the new data obtained in germination tests performed for the six mutants described in this article. Also shown below are three independent experiments (2 replicates each), in which these mutants were assayed.

**Figure:**
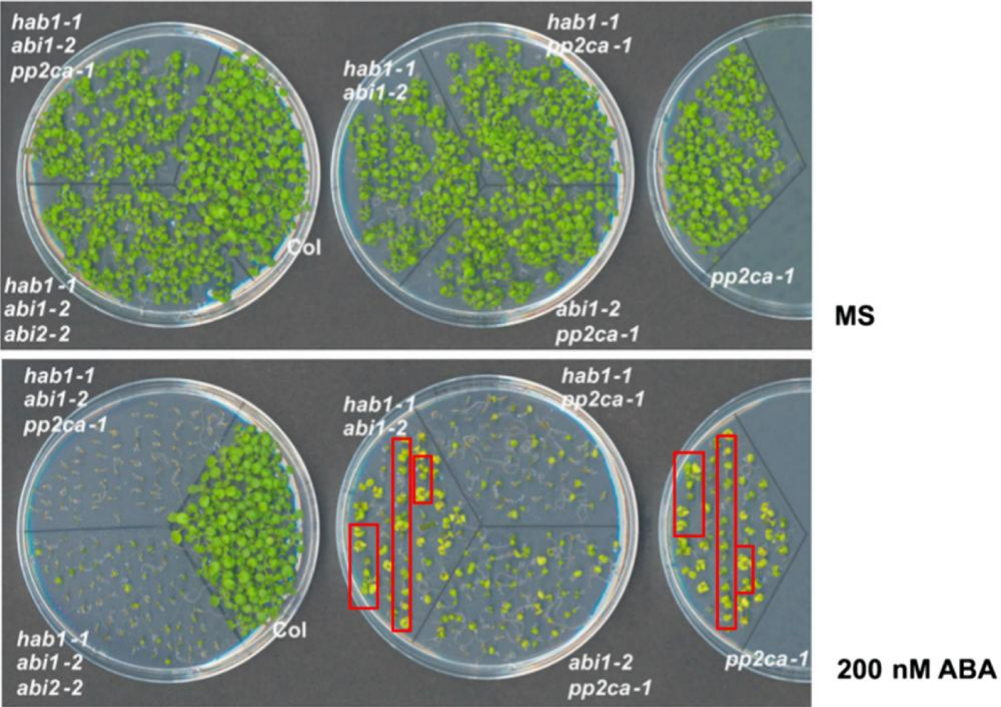


**Figure:**
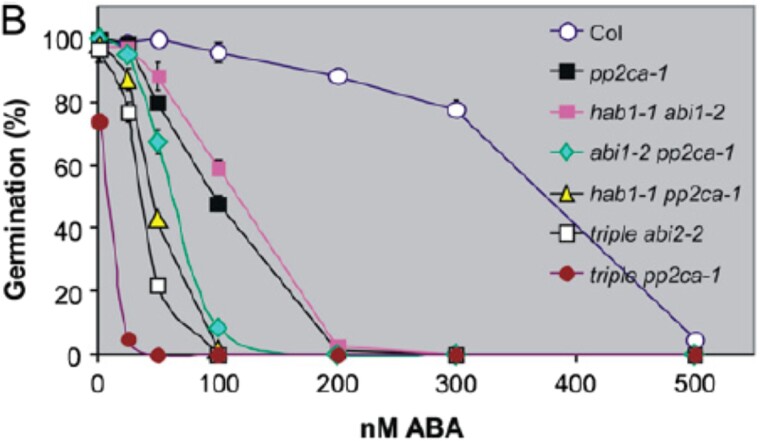


**Figure:**
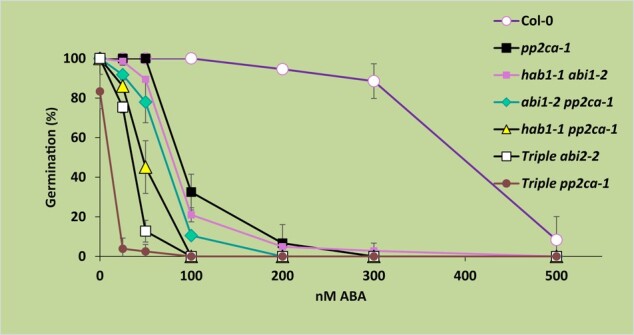


**Figure:**
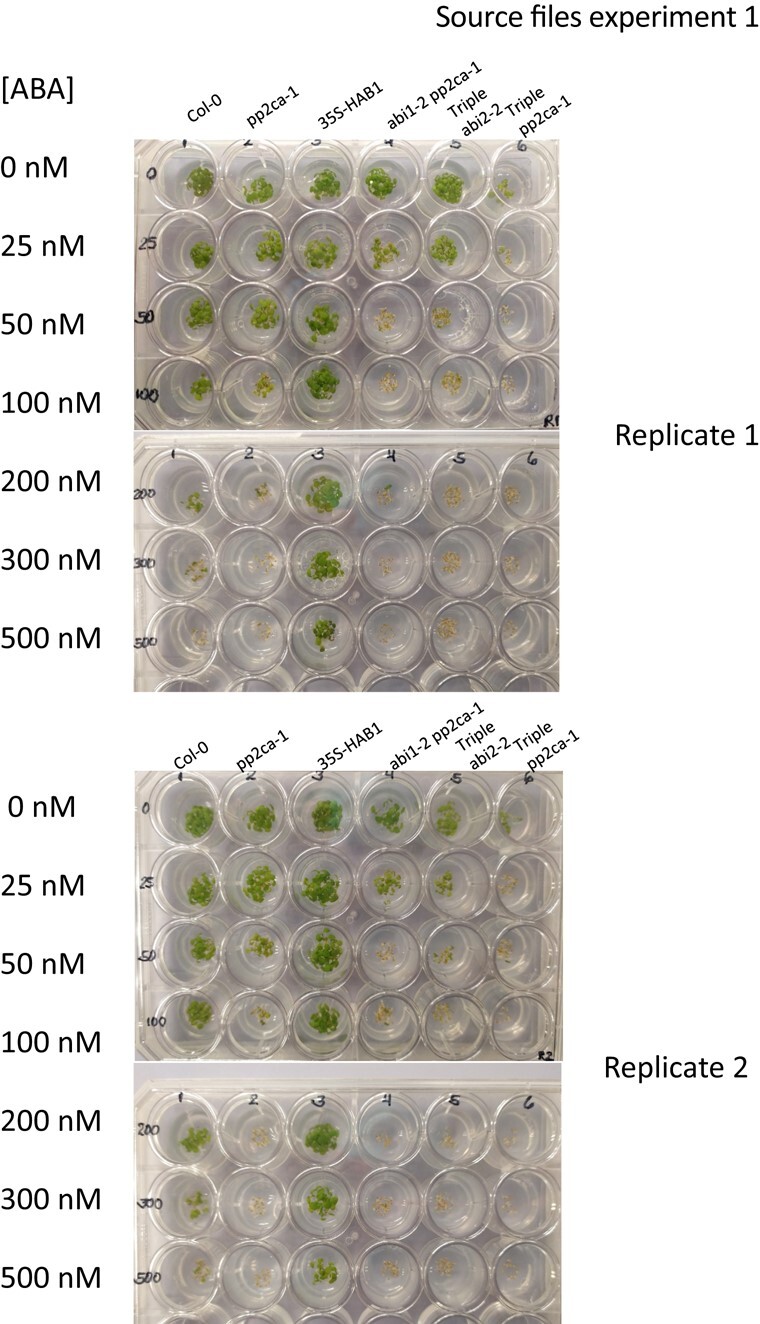


**Figure:**
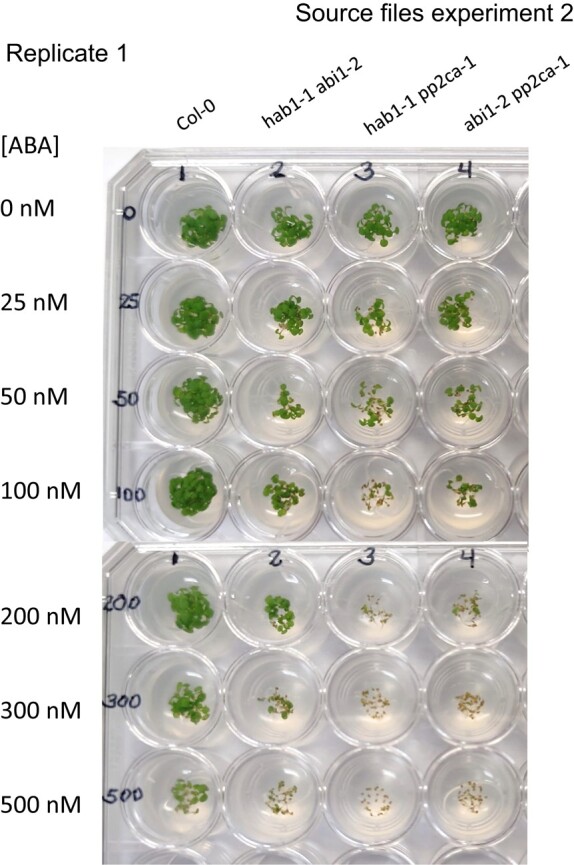


**Figure:**
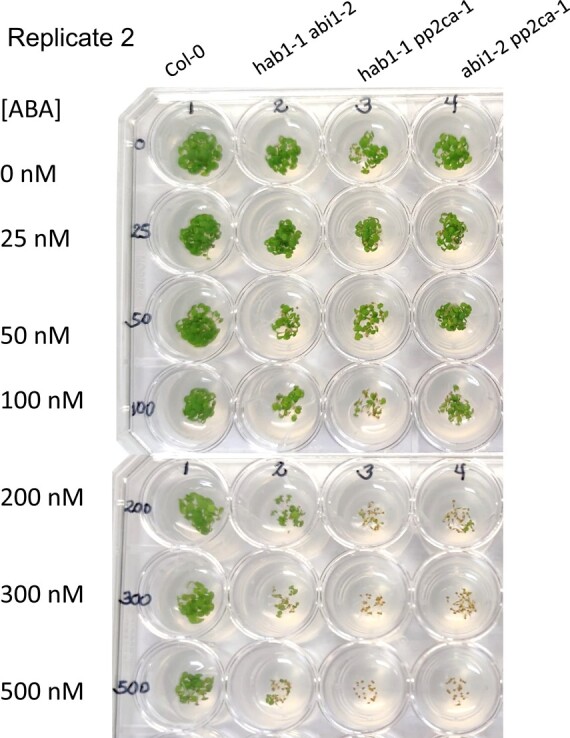


**Figure:**
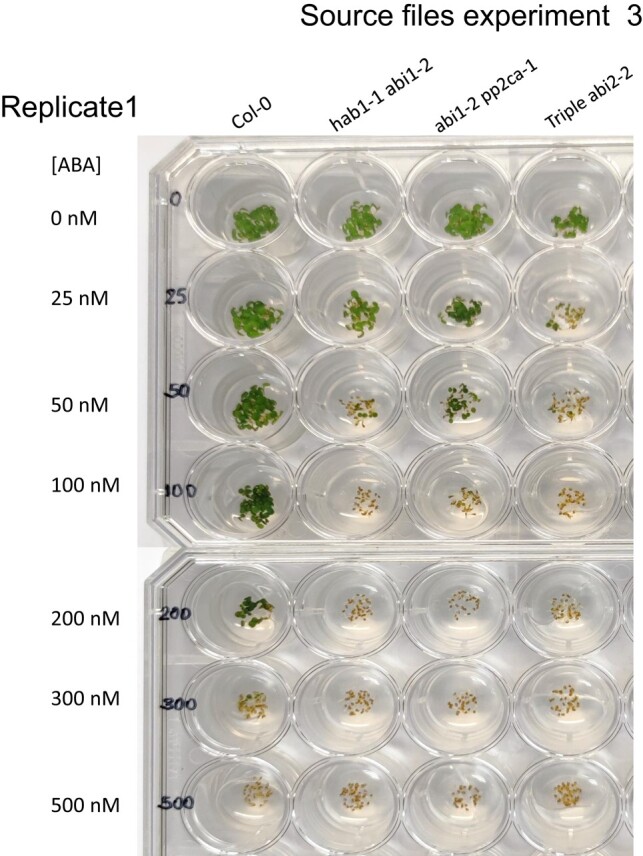


**Figure:**
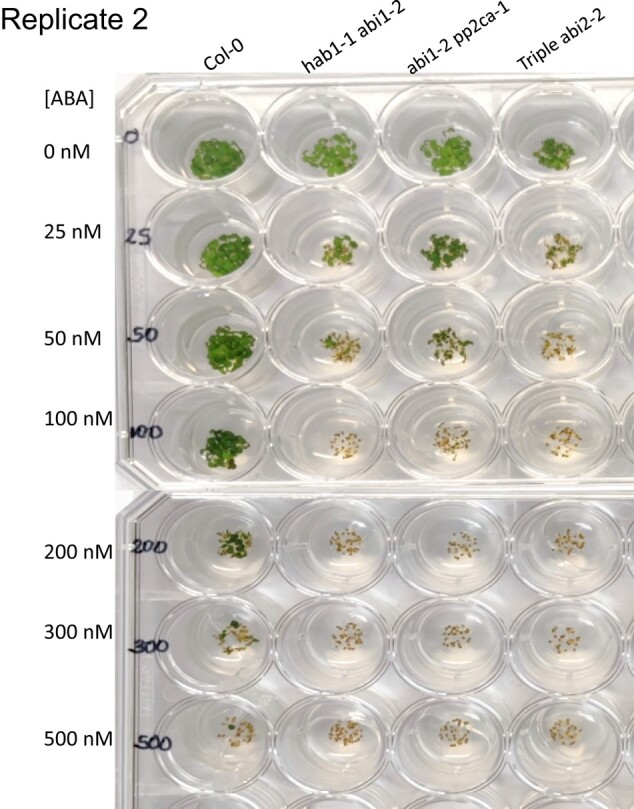


These details have been corrected only in this correction notice to preserve the published version of record.

